# Supplementary material for: Learning curve and surgical outcome of robotic assisted colorectal surgery with ERAS program
Source: Sci Rep. 2022 Nov 29;12:20566. doi: 10.1038/s41598-022-24665-w (PMC9709162; doi:10.1038/s41598-022-24665-w)
Supplement: Supplementary file 1 — Supplementary Information 1. [file 41598_2022_24665_MOESM1_ESM.zip › LSC Raw data-Ñ[▒K/068/H_ñw╝╨░O▒Kñσ.pdf]

正式摘要(電子簽章：00000018623445S)		
W52 / W52 ☐ 英文標題(注意:無英文姓名-請補病人基本檔!)	☐【讀取】憑證	

臺中榮民總醫院
出院病歷摘要

轉入號 : 轉出號 :

[redacted]

(8)流水編號	
(9)入院日期	109/02/13 (2020) 直腸外科 W52 - 153
(10)轉科日期	
(11)出院日期	109/02/20 (2020) 住院天數計 7 日

診斷

- 入院:Ca of sigmoid colon, 30 FAV
- 出院:Ca of sigmoid colon, 30 FAV, s/p Lapa AR on 2020/02/14

主訴

- incidental findings of colon tumor

病史

- This is a 45 year old male patient with history of
  1. Gout under ocassionaly use of medication
  According to the patient, he went for health examination for chest tightness first. However, incidental findings of colon tumor. He came to our CRS OPD for 2nd opinion. Colonoscopy was arranged and tumor was confirmed, Tatoo was applied. Under the impression of Colon cancer, 30cm FAV, the patient was admitted to our ward for schedule surgery.

體檢發現

- Anal inspection:
  Recto digital examination:
  Sigmoidoscopy:30CM FAV, TATTO +
  Anoscopy:
  Abdomen:
  - General appearance :
    - easy looking , conscious clear , without cardiopulmonary distress
    - Vital signs自動帶入date 體溫(BT) 脈搏(P) 呼吸(R) 血壓(BP) 身高(BH) 體重(BW) BMI
      20200213 36.5℃ 75次/分 19次/分 134/84mmHg 181cm 106kg 32.4
  - HEENT :
    - Eyes: no anemic conjunctiva, no icteric sclera, normal EOM
    - ENT: no otorrhea, no rhinorrhea, no erythematous mucosa, no injected tonsil, no deformity
  - Neck: supple, freely movable, trachea midline,no deformity, no engorged jugular vein , no palpable thyroid or lymph node enlargement
  - Chest:
    - Inspection: Grossly normal, symmetric and fully expansion, no accessory muscle used, no operation scar, no spider angiomata, no gynecomastia
    - Palpation: no palpable mass or nodule, no subcutaneous emphysema, no tenderness
    - Ausculation: clear breathing sound
  - Heart:
    - Percussion: normal heart size
    - Ausculation: regular heart beat, normal S1, S2, no gallop, no murmur
  - Abdomen:
    - Inspection: in
      - flat contour, symetrically, no scar, no stria, no dilated veins, no rashes
    - Ausculation: normoactive bowel sound, no abdominal bruits

-
  - palpation: soft, no muscle guarding or rigidity, no local tenderness, no rebounding pain, no palpable mass, no palpable pulsation
  - Percussion: no shifting dulllness, no hepatomegaly, no splenomagaly
-

Peripheral pulsation :	CA	BA	RA	FA	PA	PTA	DPA
Right side :	++	++	++	++	++	++	++/+++
Left side :	++	++	++	++	++	++	++/+++

- Genito-anus: no tenderness or palpable mass lesion or rectal shelf, normal external genitalia, normal anal tone, brown stool

CRS CONDITION

-
  - Lung

手術日期及方法

- 手術日期及方法20200214 Laparoscopic Anterior resection and anastomosis - malignant

住院治療經過

- After the admission, the patient received surgery on 2020/02/14. The patient tolerated it well and recover smoothly. The patient was discharged on 2020/02/20 under relative stable condition.

合併症

- nil

一般檢查

- 累積報告 :

DATE	TIME	ALB	TP	BIL.T	BIL.D	ALKP	AST	ALT	LDH	CHOL	TG	HDL-C	LDL	UA	NH3	AMY
1090213	2156	4.2	6.9	0.3	0.1	96	26	48	155							
1090214	0926									172	78	47	113	8.8		
1090214	1709	3.6		0.6	0.2		26	43								
1090217	0937	3.2		0.5	0.2		14	15								

DATE	TIME	WBC	RBC	HGB	HCT	MCV	PLT	BLST	NEUT	BAND	SEG	LYM	MON	EOS	BAS
1090213	2157	7610	5.05	15.6	45.3	89.7	252		62.5			24.6	5.4	6.6	0.9
1090214	1709	10530	4.78	14.8	42.9	89.7	211		87.6			8.4	2.9	0.8	0.3
1090217	0906	9870	4.64	14.7	41.6	89.7	211				79.0	13.7	6.1	1.1	0.1

DATE	TIME	NA	K	CL	CA	BUN	CREAT	Mg	P	CRP	ESR	PCT	LIPASE	LACTATE
1090213	2156	141	4.0	108	9.0	17	1.29							
1090214	0926									0.126				
1090214	1709	141	3.8	107	8.3	13	1.27							
1090217	0722													5.4
1090217	0937	139	4.2	105	8.0	10	1.24							

DATE	TIME	GLUCOSE	PAB	TRF	ZN	CU	KETONE	CK	CKMB	PT-P	PT-C	APTT-P	APTT-C	TRO-T
1090213	2156									9.9	10.1	25.9		

- 放射線報告 :檢驗項目 **:CHEST PA VIEW** 報告日期 **:1090217**
  主　訴：
  Result:
  Finding:
  > Heart size within normal limits
  > Mild increased bilateral bronchovascular markings
  > The visible bony structure appears intact
  Impression:
  As above description.

特殊檢查

病理報告

其它

出院時情況

- 3.改門診治療

出院指示

藥名	劑量	劑量單位	用法	頻次	數量	數量單位	現況	天數	單位
EM Voren cap 50mg	1	CAP	PO	BIDPRN	6	CAP	TH	3	天
(口)Takepron OD tab 30mg	1	TAB	PO	QDPRN	3	TAB	TH	3	天
Lactam tab 500mg	1	TAB	PO	Q6HPRN	12	TAB	TH	3	天

出院計劃

主治醫師 2062F 蔣鋒帆 / 住院醫師 2328K 邱俊凱

正式摘要**(電子簽章：000000186234458)**

W52 / W52

doctorRank:B
